# Surgical outcomes from nationwide implementation of the international best practice for locally advanced pancreatic cancer (PREOPANC-4) study

**DOI:** 10.1093/bjs/znag075

**Published:** 2026-06-22

**Authors:** Thomas F Stoop, Leonard W F Seelen, Freek R van ‘t Land, Jacobien C M Scheepens, Mahsoem Ali, Anna C van der Hout, B Marion van der Kolk, Bert A Bonsing, Daan J Lips, Eric R Manusama, François E J A Willemsen, Freek Daams, Geert Kazemier, Gijs A Patijn, Ignace H de Hingh, Jan H Wijsman, Jennifer Schreinemakers, Joris I Erdmann, J Sven D Mieog, Joost M Klaase, Judith A C Rietjens, Koop Bosscha, Lysanne P M Beuk, Maarten W Nijkamp, Marcel den Dulk, Marnix P M Kop, Mike S L Liem, Misha Luyer, Martijn W J Stommel, Olivier R Busch, Sebastiaan Festen, Stefan Bouwense, Tom M Karsten, Tom W van Ravens, Ulff P Neumann, Vincent E de Meijer, Vincent B Nieuwenhuijs, Werner A Draaisma, Wouter Derksen, Thomas L Bollen, Bas Groot Koerkamp, Casper H J van Eijck, I Quintus Molenaar, Christopher L Wolfgang, Marco Del Chiaro, Matthew H G Katz, Thilo Hackert, Johanna W Wilmink, Hjalmar C van Santvoort, Roeland F de Wilde, Marc G Besselink, the Dutch Pancreatic Cancer Group, Adriënne te Riele, Aniek Vlijm, Ankie van der Velden, Anne M E Bruynzeel, Anne M Stiggelbout, Anne-Marie Dietvorst, Annemarie S B Conijn-Mensink, Annebeth Haringhuizen, Anneke Roeterdink, Annette van Zweeden, Anouk van Asseldonk, Ammar A Javed, André Sterk, Annuska Schoorlemmer, Anja Stam, Aram van Brussel, Barbara M Zonderhuis, Bas Boekestijn, Bibi Martens, Brigitte Haberkorn, C Yung Nio, Celine Leenen, Daud Allajar, Deborah Reijnders-Tromp, Dirk-Jan de Groot, Els Wink-Van Gestel, Emile D Kerver, Eran van Veldhuisen, Eva Versteijne, Evelien van Alphen, Evert van den Broek, Geert A Cirkel, Geertjan van Tienhoven, Geeske Heringa, Gert Stockmans, Gian Piero Serafino, Hannàh N Lelieveld-Rier, Hanneke W M van Laarhoven, Hanneke Zuetenhorst, Haske van Veenendaal, Hetty van Mierlo, Ingeborg Griffioen, Ingmar F Rompen, Irene E G van Hellemond, Iryna Symarska, Jacques Peters, Jan-Willem B de Groot, Jeanette Ham, Jeroen Willems, Jessica Coes, Joachim Kikomeko, Johan van Rooijen, Joanne Verheij, Joost Nederend, Mirte Streppel, José Schellekens, Kishan R D Lutchman, Judith de Vos-Geelen, Laura R Moolenaar, Laurens van der Waaij, Laurens Beerepoot, Leonie J Mekenkamp, Lilly H J Brada, Lobke van Leeuwen—Snoeks, Lodewijk Brosens, Linda Garms, Leonieke W Kranenburg, Loes J Peters, Maartje Los, Marco B Polée, Marjolein Y V Homs, Marit Suttorp, Marieke S Walma, Marjon Oostdijk, Marleen Duizer, Melanie F M A Marting, Michael Doukas, Miriam L Wumkes, Monica Seijbel, Nicole D Hildebrand, Nynke M Michiels, Olaf Loosveld, Peter Nieboer, Ramon Bax, Roel Haen, Roel Heesakkers, Ronald M van Dam, Rosa van der Werf, Rowan van Gerven, Rune van de Wetering, Rutger T Theijse, Ruud Blankenburgh, Sandra Bakker, Sanne Achten, Saskia A C Luelmo, Simone Augustinus, Simone Rötgerink, Stijn Crobach, K C Khe Tran, Tess M E van Ramshorst, Wendy van der Deure-Gielisse, Wouter W te Riele

**Affiliations:** Department of Surgery, Amsterdam UMC, location University of Amsterdam, Amsterdam, The Netherlands; Cancer Centre Amsterdam, Amsterdam, The Netherlands; Division of Surgical Oncology, Department of Surgery, University of Colorado Anschutz Medical Campus, Aurora, Colorado, USA; Department of Surgery, Regional Academic Cancer Centre Utrecht, University Medical Centre Utrecht/St Antonius Hospital Nieuwegein, Utrecht & Nieuwegein, The Netherlands; Department of Surgery and Pulmonology, Erasmus MC Cancer Institute, University Medical Centre, Rotterdam, The Netherlands; Department of Surgery, Regional Academic Cancer Centre Utrecht, University Medical Centre Utrecht/St Antonius Hospital Nieuwegein, Utrecht & Nieuwegein, The Netherlands; Cancer Centre Amsterdam, Amsterdam, The Netherlands; Department of Surgery, Amsterdam UMC, location University of Amsterdam, Amsterdam, The Netherlands; Department of Public Health, Erasmus MC, University Medical Centre Rotterdam, Rotterdam, The Netherlands; Department of Surgery, Radboud University Medical Centre, Nijmegen, The Netherlands; Department of Surgery, Leiden University Medical Centre, Leiden, The Netherlands; Department of Surgery, Medisch Spectrum Twente, Enschede, The Netherlands; Department of Surgery, Medical Centre Leeuwarden, Leeuwarden, The Netherlands; Department of Radiology, Erasmus MC, University Medical Centre Rotterdam, Rotterdam, The Netherlands; Cancer Centre Amsterdam, Amsterdam, The Netherlands; Department of Surgery, Amsterdam UMC, location University of Amsterdam, Amsterdam, The Netherlands; Cancer Centre Amsterdam, Amsterdam, The Netherlands; Department of Surgery, Amsterdam UMC, location University of Amsterdam, Amsterdam, The Netherlands; Department of Surgery, Isala, Zwolle, The Netherlands; Department of Surgery, Catharina Hospital, Eindhoven, The Netherlands; Department of Surgery, Amphia Ziekenhuis, Breda, The Netherlands; Department of Surgery, Amphia Ziekenhuis, Breda, The Netherlands; Department of Surgery, Amsterdam UMC, location University of Amsterdam, Amsterdam, The Netherlands; Cancer Centre Amsterdam, Amsterdam, The Netherlands; Department of Surgery, Leiden University Medical Centre, Leiden, The Netherlands; Department of Surgery, University of Groningen and University Medical Centre Groningen, Groningen, The Netherlands; Department of Public Health, Erasmus MC, University Medical Centre Rotterdam, Rotterdam, The Netherlands; Department of Design, Organization, and Strategy, Faculty of Industrial Design Engineering, Delft University of Technology, Delft, The Netherlands; Department of Surgery, Jeroen Bosch Hospital, ‘s Hertogenbosch, The Netherlands; Department of Surgery and Pulmonology, Erasmus MC Cancer Institute, University Medical Centre, Rotterdam, The Netherlands; Department of Surgery, University of Groningen and University Medical Centre Groningen, Groningen, The Netherlands; Department of Surgery, Maastricht University Medical Centre, Maastricht, The Netherlands; Nutrim School for Nutrition and Translational Research in Metabolism, Maastricht, The Netherlands; Cancer Centre Amsterdam, Amsterdam, The Netherlands; Department of Radiology, Amsterdam UMC, location University of Amsterdam, Amsterdam, The Netherlands; Department of Surgery, Medisch Spectrum Twente, Enschede, The Netherlands; Department of Surgery, Catharina Hospital, Eindhoven, The Netherlands; Department of Surgery, Radboud University Medical Centre, Nijmegen, The Netherlands; Department of Surgery, Amsterdam UMC, location University of Amsterdam, Amsterdam, The Netherlands; Cancer Centre Amsterdam, Amsterdam, The Netherlands; Department of Surgery, OLVG, Amsterdam, The Netherlands; Department of Surgery, Maastricht University Medical Centre, Maastricht, The Netherlands; Nutrim School for Nutrition and Translational Research in Metabolism, Maastricht, The Netherlands; Department of Surgery, OLVG, Amsterdam, The Netherlands; Department of Surgery, Leiden University Medical Centre, Leiden, The Netherlands; Nutrim School for Nutrition and Translational Research in Metabolism, Maastricht, The Netherlands; Department of Surgery, University Hospital Essen, Essen, Germany; Department of Surgery, University of Groningen and University Medical Centre Groningen, Groningen, The Netherlands; Department of Surgery, Isala, Zwolle, The Netherlands; Department of Surgery, Jeroen Bosch Hospital, ‘s Hertogenbosch, The Netherlands; Department of Surgery, Regional Academic Cancer Centre Utrecht, University Medical Centre Utrecht/St Antonius Hospital Nieuwegein, Utrecht & Nieuwegein, The Netherlands; Department of Radiology, St Antonius Hospital, Nieuwegein, The Netherlands; Department of Surgery and Pulmonology, Erasmus MC Cancer Institute, University Medical Centre, Rotterdam, The Netherlands; Department of Surgery and Pulmonology, Erasmus MC Cancer Institute, University Medical Centre, Rotterdam, The Netherlands; Department of Surgery, Regional Academic Cancer Centre Utrecht, University Medical Centre Utrecht/St Antonius Hospital Nieuwegein, Utrecht & Nieuwegein, The Netherlands; Department of Surgery, NYU Langone Health, NewYork, New York, USA; Division of Surgical Oncology, Department of Surgery, University of Colorado Anschutz Medical Campus, Aurora, Colorado, USA; Department of Surgical Oncology, The University of Texas MD Anderson Cancer Center, Houston, Texas, USA; Department of General, Visceral, and Thoracic Surgery, University Hospital Hamburg-Eppendorf, Hamburg, Germany; Cancer Centre Amsterdam, Amsterdam, The Netherlands; Department of Medical Oncology, Amsterdam UMC, location University of Amsterdam, Amsterdam, The Netherlands; Department of Surgery, Regional Academic Cancer Centre Utrecht, University Medical Centre Utrecht/St Antonius Hospital Nieuwegein, Utrecht & Nieuwegein, The Netherlands; Department of Surgery and Pulmonology, Erasmus MC Cancer Institute, University Medical Centre, Rotterdam, The Netherlands; Department of Surgery, Amsterdam UMC, location University of Amsterdam, Amsterdam, The Netherlands; Cancer Centre Amsterdam, Amsterdam, The Netherlands

## Abstract

**Background:**

In expert centres, surgical resection rates for patients with locally advanced pancreatic cancer (LAPC) after induction chemotherapy have increased beyond 20% with subsequent 25% 5-year overall survival (OS). In the Netherlands, however, the historical low 8% LAPC resection rate compared with 23% in international expert centres reflects a relative reluctance to perform resections. Thereby, opportunities to achieve long-term survival in appropriately selected patients may be missed. The aim of this study was to evaluate whether nationwide implementation of international multidisciplinary best practice for LAPC management is feasible while maintaining surgical safety benchmarks (in-hospital/30-day major morbidity <50% and mortality ≤5%).

**Methods:**

A multidisciplinary protocol was designed in collaboration with four international experts and prospectively implemented nationwide within the Dutch Pancreatic Cancer Group (DPCG) (2022–2024). This observational cohort included consecutive patients diagnosed with LAPC, defined by DPCG criteria. Eligible patients had radiologically non-progressive disease after ≥4 months of multiagent chemotherapy. All patients who underwent resection were included in this safety analysis. A predefined subgroup analysis included patients with National Comprehensive Cancer Network (NCCN) LAPC. Primary outcomes included in-hospital/30-day major morbidity (that is Clavien–Dindo grade ≥IIIa) and mortality. The expected number of resections was 53.

**Results:**

Overall, 180 patients with LAPC underwent surgical exploration, of whom 155 (86%) underwent resection in 11 centres. Most resections (74%) were performed in the three LAPC surgical centres. An extended resection was performed for 77% of patients, including portomesenteric venous (60%), multivisceral (23%), and arterial (21%) resections. The in-hospital/30-day major morbidity rate was 44% and the mortality rate was 0.6%, both within pre-established safety benchmarks. Benchmarks were also reached for patients with NCCN LAPC (49% major morbidity and 2% mortality).

**Conclusion:**

Nationwide implementation of the international best practice for LAPC was feasible with nearly three times more resections performed than expected, while morbidity and mortality remained well within predefined safety benchmarks.

## Teaser

Nationwide implementation of the international best practice for locally advanced pancreatic cancer (LAPC) surgery met the predefined benchmarks for surgical safety with 44% major morbidity and 0.6% mortality.

## Introduction

In the past decade, the use of modern multiagent chemotherapy for localized pancreatic adenocarcinoma facilitated an ongoing paradigm shift towards a biology-driven treatment approach. This has improved opportunities for patients with locally advanced pancreatic cancer (LAPC), as shown by several international expert centres^1^. Due to better disease control with multiagent chemotherapy and subsequent response evaluation criteria using anatomy-, biology-, and condition-related (that is A, B, and C) parameters^2–4^, some 23% (95% c.i. 11% to 40%) of patients with LAPC can now undergo surgical resection with acceptable complication rates and 5-year OS of 25% in expert centres^5–11^.

However, a recent nationwide observational study demonstrated a resection rate of 8% for patients with LAPC treated with induction chemotherapy in the Netherlands, despite acceptable surgical and survival outcomes^12,13^. The substantial difference in resection rates between the international literature and the Netherlands (23% *versus* 8%) cannot be explained by referral and publication bias alone, considering the more restrictive Dutch resectability criteria compared with the international guidelines. Instead, differences and variation in approaches about induction therapy, patient selection, and surgical experience and techniques are more likely explanations^12,13^.

This implies that there is room for improvement in the multidisciplinary treatment of patients with LAPC in the Netherlands. Therefore, a nationwide implementation trial was initiated to improve the multidisciplinary care of patients with LAPC through standardization of oncological and surgical care^14^. This programme implemented the international best practice for LAPC in the Netherlands in close collaboration with four international experts, aiming to improve overall survival (OS) in a patient-centred manner while maintaining surgical safety. The aim of this study was to investigate whether the benchmarks for safety after surgical resection were met within the PREOPANC-4 implementation study.

## Methods

This nationwide prospective observational PREOPANC-4 implementation study is reported in accordance with the STROBE guidelines^15^. The Amsterdam UMC medical ethical committee evaluated the study protocol and concluded that the Medical Research Involving Human Subjects Act (WMO) does not apply (W21_487 # 21.541). In addition, the local medical ethical committee from each participating centre approved their participation. The methodology applying to the surgical safety after resection in the PREOPANC-4 implementation study is summarized below. More details about the methodology including the training programme and clinical protocol including the standardized care strategy can be found in *Appendix S1* and the published study protocol (NCT05524090)^14^. A nationwide online twice-weekly LAPC Expert Panel was installed where patients for surgical exploration would be discussed.

### Study design

The PREOPANC-4 prospective observational cohort study comprised training and implementation phases. The training phase started in 2021, after which the 3-year implementation was conducted from January 2022 to December 2024. All 15 centres from the Dutch Pancreatic Cancer Group (DPCG) participated in this trial, comprising all pancreatic surgery-certified centres in the Netherlands^16^. Four international experts in the field of LAPC surgery from the USA (C.L.W., M.D.C., and M.H.G.K.) and Germany (T.H.) oversaw the training programme, co-developed the clinical protocol, and proctored during the implementation.

Consecutive adult patients (that is patients aged ≥18 years) diagnosed with pathology-confirmed LAPC following the DPCG criteria (that is arterial involvement >90° and/or portomesenteric venous >270° involvement or occlusion) based on cross-sectional imaging at the time of diagnosis were considered eligible to receive care according to the designed clinical workup. See *Appendix S2* for the resectability criteria. This clinical protocol was a dynamic process that was further developed based on new insights and evolving evidence. Modifications were made to the clinical protocol during the implementation phase, as described in *Appendix S3*.

The implementation was prospectively monitored by the registration of all consecutive patients with non-progressive disease based on cross-sectional imaging following Response Evaluation Criteria In Solid Tumours (RECIST) (version 1.1) after ≥4 months of chemotherapy with (m)FOLFIRINOX (that is a combination of 5-fluorouracil with leucovorin, irinotecan, and oxaliplatin) and/or gemcitabine-nab-paclitaxel^17^.

In the present study, the surgical outcomes of all patients who underwent surgical resection within the PREOPANC-4 cohort were evaluated to investigate whether the predefined benchmarks for surgical safety for the primary endpoints in-hospital/30-day major morbidity (<50%) and in-hospital/30-day mortality (≤5% mortality) were met. These benchmarks were established by the protocol group, based on the international benchmarks for pancreatoduodenectomy with portomesenteric venous resection and the additional risks associated with arterial resections^18,19^. Based on a historical Dutch LAPC cohort, it was calculated that 53 LAPC resections would be performed during the 3-year implementation interval^12^.

Additional surgical outcomes are reported, including the 90-day mortality rate from the date of surgery. The outcomes are stratified for patients diagnosed with LAPC according to the National Comprehensive Cancer Network (NCCN) criteria^20,21^ and for types of surgical procedures including the type of pancreatectomy (that is pancreatoduodenectomy, left-sided pancreatic resection, or total pancreatectomy) and (the type of) extended resection (that is arterial resection, portomesenteric venous resection, or multivisceral resection) according to the Heidelberg classification^22–24^.

### Surgical principles

The three highest volume centres for pancreatic surgery including LAPC surgery (that is Amsterdam UMC, Erasmus MC, and RACU) functioned as referral centres for LAPC surgery during the implementation. The four other university medical centres (that is UMCG, MUMC+, LUMC, and Radboud UMC) participated in the training programme, working towards a situation in the Netherlands where LAPC surgery is concentrated in five centres^13^. The Johns Hopkins LAPC score (that is 1, 2, or 3) was used to grade the complexity of tumours, which supported the process of referring patients to the three referral centres in the case of highly complex tumours^25^. Based on the surgical training programme, the specific surgical principles were followed during the implementation phase, as described in *Appendix S1*^26–41^.

### Definitions

Patient performance and co-morbidity status at the time of diagnosis were defined following the Eastern Cooperative Oncology Group (ECOG) classification and the age-adjusted Charlson Co-morbidity Index. Additionally, patient performance status at the time of surgery was classified according to the ASA. Imaging-based resectability status was classified according to the resectability criteria from the DPCG and the NCCN (version 2.2024), presented in *Appendix S2*^20,42^. The types of pancreatic resection and extended resection were defined according to the International Study Group for Pancreatic Surgery (ISGPS)^21^. Resection of the common hepatic artery (CHA) as part of a coeliac axis resection was not counted separately as hepatic artery resection.

Pancreatic surgery-specific complications, including postoperative pancreatic fistula (POPF), post-pancreatectomy hemorrhage (PPH), delayed gastric emptying, chyle leak, and bile leak, were classified following the ISGPS and International Study Group of Liver Surgery (ISGLS) guidelines, with grades B and C considered to be clinically relevant^43–47^. Organ failure was defined as meeting at least one of the following criteria: ≥24 h of respiratory insufficiency requiring intubation, ≥24 h of haemodynamic instability requiring inotropics, and/or ≥24 h of renal insufficiency requiring dialysis. In-hospital/30-day major morbidity was defined as Clavien–Dindo grade ≥IIIa^48^. Readmission was defined as a hospital admission that involved a stay of at least one night that occurred within 90 days after index surgery. The data were collected by an experienced clinical researcher and medical doctor (T.F.S.)^49^.

## Results

Overall, 180 patients underwent surgical exploration with intention for resection, of whom 155 patients (86%) underwent resection. Of these 180 patients, 92 patients (51%) were discussed by the twice-weekly national LAPC Expert Panel. Reasons for not performing a resection were metastatic disease (14 patients (8%)) and extensive tumour involvement (11 patients (6%)) including arterial involvement (9 patients (5%)), portomesenteric venous involvement (5 patients (3%)), and/or involvement of multivisceral structures (1 patient (<1%)).

Surgery started with a staging laparoscopy for 132 patients (73% (missing data for 2 patients)) with a 4% yield of detecting occult metastases. After a negative staging laparoscopy for 127 patients, an extra 4 patients (3%) were found to have occult metastases during laparotomy. For the 173 patients who underwent an explorative laparotomy, intraoperative ultrasonography (IOUS) was used for 51 patients (29% (missing data for 2 patients)) to assess potential liver lesions (15 patients (9%)) and/or vascular involvement (50 patients (29%)). IOUS changed the surgical management for 11 patients (22%), based on the detection of liver metastases (1 patient (2%)) or the extent of vascular involvement (10 patients (20%)). For the latter ten patients, IOUS revealed less vascular involvement for nine patients (18%) and more vascular involvement for one patient (2%) than was expected before IOUS.

Two patients decided to undergo surgical exploration outside of the Netherlands. One patient underwent surgical exploration abroad, in which metastatic disease was found intraoperatively. For the second patient, surgery was recommended by the LAPC Expert Panel, but the patient underwent resection abroad. The surgical outcome for this patient who underwent resection was not included in this study.

### Baseline characteristics and induction therapy

At diagnosis, the median age was 63 (interquartile range (i.q.r.) 57 to 70) years, most patients had an ECOG performance status of 0–1 (132 patients (86%)), and the median Charlson Co-morbidity Index was 3 (i.q.r. 2 to 4). Most tumours were located in the pancreatic head (118 patients (77%)) and 37% of patients met the NCCN resectability criteria for LAPC. See *Table 1* for more details about baseline characteristics and *Appendix S5* for the vascular involvement at diagnosis.

**Table 1:** Baseline characteristics

Variables	Values
**Patients, *n***	**154**
Age (years), median (i.q.r.)	63 (57–70)
Female	79 (51)
BMI (kg/m^2^), median (i.q.r.)	24 (22–26)
Missing	2 (1)
ECOG performance status	
0	63 (41)
1	69 (45)
≥2	8 (5)
Missing	14 (9)
Charlson Co-morbidity Index, median (i.q.r.)	3 (2–4)
**Disease characteristics**	
Tumour location	
Head	118 (77)
Body/tail (left-sided)	36 (23)
Tumour size (mm), median (i.q.r.)	35 (29–44)
Unknown/not possible to assess	5 (3)
NCCN LAPC	57 (37)
CA19-9 (units/ml), median (i.q.r.)	262 (54–886)
Missing	1 (<1)
CEA (ng/ml), median (i.q.r.)	3.0 (2.1–5.9)
Missing	53 (34)
**Induction therapy**	
First-line chemotherapy	
(m)FOLFIRINOX	144 (94)
Cycles, median (i.q.r.)	8 (8–10)
Gemcitabine-nab-paclitaxel	5 (3)
Cycles, median (i.q.r.)	4 (4–6)
Gemcitabine	5 (3)
Cycles, median (i.q.r.)	3 (2–3)
External beam radiotherapy	3 (2)
Dose reduction	125 (81)
Missing	3 (2)
Chemotherapy switch	13 (8)
(m)FOLFIRINOX	5 (38)
Cycles, median (i.q.r.)	8 (8–9)
Gemcitabine-nab-paclitaxel	8 (62)
Cycles, median (i.q.r.)	4 (2–4)
Dose reduction	10 (77)
Missing	1 (8)
**Restaging**	
RECIST	
Stable disease	81 (53)
Partial response	71 (46)
Complete response	1 (<1)
Missing/not possible to assess	1 (<1)
CA19-9 (units/ml), median (i.q.r.)	41 (15–77)
Missing	3 (2)
CA19-9 relative change, median (i.q.r.)*	−88% (−94% to −75%)
CEA (ng/ml), median (i.q.r.)	3.2 (2.0–4.8)
Missing	68 (44)

Values are *n* (%) unless otherwise indicated. *For patients with serum CA19-9 ≥37 units/ml at diagnosis. i.q.r., interquartile range; ECOG, Eastern Cooperative Oncology Group; NCCN, National Comprehensive Cancer Network; LAPC, locally advanced pancreatic cancer; CA19-9, carbohydrate antigen 19-9; CEA, carcinoembryonic antigen; (m)FOLFIRINOX, a (modified) combination of 5-fluorouracil with leucovorin, irinotecan, and oxaliplatin; RECIST, Reseponse Evaluation Criteria In Solid Tumours.

The most used induction chemotherapy regimen was (m)FOLFIRINOX (144 patients (94%)). Five patients (3%) were initially diagnosed with borderline resectable pancreatic cancer, but progressed to LAPC during neoadjuvant therapy with gemcitabine plus external beam radiotherapy. Thereafter, these patients followed the PREOPANC-4 clinical protocol including ≥4 months of induction therapy with (m)FOLFIRINOX or gemcitabine-nab-paclitaxel. In total, 13 patients (8%) underwent a switch in chemotherapy, mainly to gemcitabine-nab-paclitaxel (8 patients (62%)). See *Table 1* for details about induction therapy regimens.

At final restaging, mostly RECIST stable disease (53%) or partial response (46%) was seen. For patients with elevated serum CA19-9 levels at diagnosis (that is ≥37 units/ml), a median relative reduction in serum CA19-9 of −88% (i.q.r. −94% to −75%) was seen. See *Table 1* for more information about preoperative disease characteristics after induction therapy.

### Surgical procedures

Overall, resections were performed in 11 centres and 74% of resections were performed in the three LAPC referral centres. Pancreatoduodenectomy was the most common procedure (113 patients (73%)). An extended resection was performed for 77% of patients, including portomesenteric venous resection (92 patients (60%)), arterial resection (32 patients (21%)), and multivisceral resection (36 patients (23%)). Arterial divestment was performed for 119 patients (77%), involving the superior mesenteric artery (SMA) (65%), hepatic artery (34%), and coeliac axis (14%). See *Table 2* and *Appendix S6* for a detailed description of the surgical procedures.

**Table 2:** Surgical details

Variables	Values
**Patients, *n***	154
Type of centre	
Referral centre (3 university medical centres)	114 (74)
Other university medical centre	34 (22)
Non-university centre	6 (4)
ASA grade	
I–II	109 (71)
III–IV	44 (29)
Missing	1 (<1)
**Procedure details**	
Pancreatectomy	
Pancreatoduodenectomy	113 (73)
Left-sided pancreatic resection	29 (19)
Total pancreatectomy	12 (8)
Arterial divestment	119 (77)
SMA	100 (65)
Hepatic artery	53 (34)
Coeliac axis	22 (14)
Extended resection	118 (77)
Vascular resection	111 (72)
Portomesenteric venous resection*	92 (60)
Type 1–2	25 (16)
Type 3–4	63 (41)
Resection without reconstruction	5 (3)
Inferior vena cava	2 (1)
Arterial resection	32 (21)
Hepatic artery	19 (12)
Reconstruction	10 (53)
Coeliac axis	14 (9)
Reconstruction	1 (7)
Superior mesenteric artery	2 (1)
Multiple arterial segment resection	3 (2)
Portomesenteric venous + arterial resection	14 (9)
Multivisceral resection	36 (23)
Stomach	5 (3)
(Meso)colon and/or its vasculature	23 (15)
Small bowel	9 (6)
Adrenal	9 (6)
Kidney and/or its vasculature	2 (1)†
Diaphragm	2 (1)
Liver	0 (0)

Values are *n* (%) unless otherwise indicated. *One patient underwent primary closure of the superior mesenteric vein (SMV) and type 2 inferior mesenteric vein (IMV) resection with bovine patch reconstruction. †Including one patient who underwent a kidney autotransplantation because of involvement of the renal artery. SMA, superior mesenteric artery.

### Surgical outcomes

The rate of in-hospital/30-day major morbidity was 44% (95% c.i. 44% to 52%) and the rate of in-hospital/30-day mortality was 0.6% (95% c.i. 0% to 4%). For patients with NCCN LAPC, the rates of major morbidity and mortality were 49% (95% c.i. 37% to 62%) and 2% (95% c.i. 0% to 9%) respectively. Details about surgical outcomes are presented in *Table 3*. Stratification of surgical outcomes by resectability criteria, type of extended resection, and type of pancreatectomy is presented in *Appendices S8–S10*.

**Table 3:** Surgical outcomes

Variables	Values
Patients, *n*	154
**Intraoperative outcomes**	
Intraoperative blood loss (ml), median (i.q.r.)	1000 (500–1525)
Missing	1 (<1)
Intraoperative blood transfusion	30 (19)*
Number of transfusions, median (i.q.r.)	2 (1–2)
Operating time (min), median (i.q.r.)	416 (325–509)
Missing	2 (1)
Macrosopically radical resection (R0/R1)	153 (99)
**Postoperative outcomes**	
Delayed gastric emptying	
Grade B	11 (7)
Grade C	15 (10)
Post-pancreatectomy haemorrhage	
Grade B	24 (16)
Grade C	4 (3)
Postoperative pancreatic fistula‡	
Grade B	18 (13)
Grade C	1 (<1)
Bile leakage§	
Grade B	8 (6)
Grade C	1 (<1)
Chyle leakage	
Grade B	18 (12)
Grade C	0 (0)
Major morbidity	68 (44)
Reoperation	15 (10)
Delayed reconstruction	2 (1)
Postoperative complication-related¶	13 (8)
Organ failure	3 (2)
Single-organ failure	2 (1)
Multi-organ failure	1 (<1)
MCU/ICU admission	13 (8)
Length of stay (days), median (i.q.r.)	5 (4–7)
Hospital stay (days), median (i.q.r.)	9 (7–16)
In-hospital/30-day mortality	1 (<1)
Ninety-day mortality	5 (3)
Readmission†	40 (26)

Values are *n* (%) unless otherwise indicated. *Missing *n* = 2. †Patients who died during admission (that is in-hospital mortality) are excluded for the denominator. ‡Patients who underwent total pancreatectomy are excluded for the denominator. §Patients who underwent left-sided pancreatectomy are excluded for the denominator. ¶See *Appendix S7* for more details about the relaparotomies. i.q.r., interquartile range; MCU, medium care unit.

## Discussion

In this nationwide study, implementation of the international best practice for LAPC was found to be feasible and safe (44% in-hospital/30-day major morbidity and 0.6% in-hospital/30-day mortality) after multidisciplinary training overseen by four international experts. The 154 resections performed during the 3-year implementation interval were nearly three times higher than anticipated.

This is the first report of a structured nationwide implementation programme for the management of LAPC. The use of LAPC surgery after induction chemotherapy has strongly expanded in expert centres around the world^50^, supported by the growing body of evidence showing promising survival outcomes^51^. This evolution within specialized centres is based on heterogeneous induction therapy protocols, surgical selection criteria, and operative techniques^52^. Meanwhile, the translation of this highly specialized care into structured and standardized care pathways at regional and national levels is highly challenging. High surgical morbidity and concern for early disease recurrence (that is one-third of patients within 6 months) cause controversy and scepticism among clinical specialists in regional centres regarding the value of surgery for these patients, possibly leading to non-referral of patients to the LAPC expert centres^53^. This is illustrated by the fact that about 24–40% of patients diagnosed with LAPC in the Netherlands and the USA do not receive any tumour-directed treatment^50,54^. Regional and national healthcare networks are, therefore, of vital importance when striving for standardization of oncological and surgical treatment, adequate patient selection, and wide implementation of this complex multidisciplinary care^55^.

The PREOPANC-4 implementation study has been a rather unique national programme that standardized the oncological and surgical treatment of patients with LAPC at a national level with the help of four international experts in the field of LAPC surgery. Naturally, variations in oncological and surgical practices existed among the four proctors. Differences included the duration of induction chemotherapy, serum CA19-9 thresholds for minimum relative response and maximum absolute values guiding surgical exploration, and surgical techniques, including indications for arterial resection and use of synthetic grafts for vascular reconstruction. Differences in practice were extensively discussed during webinars and additional meetings, after which the final clinical protocol was designed, supported by the experts and Dutch clinicians. Importantly, this strategy implemented within the PREOPANC-4 programme should be considered as a best practice rather than ‘the’ best practice, as there might be equally effective or alternative surgical and oncological strategies.

Differences can be seen between the PREOPANC-4 resection cohort and a recent historical LAPC resection cohort from the DPCG. In a 7-year interval (2014–2020), a total of 142 patients with LAPC underwent resection, of whom 49 patients (35%) had NCCN LAPC^13^. This increased to 154 resections during the 3-year PREOPANC-4 study. This indeed implies that the implementation strategy led to nationwide improvement, which is also illustrated by the numerically higher rates of arterial resection (14% to 21%) and portomesenteric venous resection (51% to 60%) for the PREOPANC-4 cohort compared with the historical cohort^13^.

About three-quarters of the resections were performed in the three LAPC referral university centres. Most other patients (22%) were operated on in the other four university centres. The seven university centres participated in the multidisciplinary and surgical training programme with the aim of eventually having five LAPC referral centres in the Netherlands. During the PREOPANC-4 implementation, three of them had a collaboration with another university centre, to enhance their volume of LAPC surgery. Only six patients underwent resection in a non-university centre, which predominantly concerned patients with radiological downstaging after induction therapy to DPCG borderline resectable disease.

Overall, a standard pancreatic resection without vascular or multivisceral resection was performed for 23% of patients. Arterial involvement based on imaging could be managed with arterial divestment without the need for an arterial resection in 77% of patients. Even when an arterial divestment seems to be sufficient before surgery^31^, referring these patients to an expert centre in LAPC surgery is crucial, as the procedure may need to be converted to an arterial resection with reconstruction due to vascular wall invasion or iatrogenic injury^56^. This is illustrated by the two patients who required an SMA resection because of iatrogenic damage caused by divestment. In at least two other patients, SMA divestment was intraoperatively complicated by an SMA dissection, which was successfully managed with immediate stenting.

The PREOPANC-4 implementation study demonstrated a safe nationwide implementation of the international standard of care for LAPC surgery in the Netherlands, executed by experienced pancreatic surgery centres. Next, indications for surgery could be gradually expanded technically within the university (referral) centres for even more rigorously selected patients, striving for further optimization of patient selection and meanwhile maintaining the surgical safety. The fact that the median length of portomesenteric venous resection was 30 mm underlines the potential room for further pushing the technical borders. The other primary and secondary endpoints from the PREOPANC-4 implementation study including survival will follow once the follow-up is completed, together with the surgical radicality, resection rate, patient-reported outcome measures, and results from the shared decision-making training programme^14,57^.

The following limitation should be considered when interpreting the present results. This study was conducted within centres from the DPCG, including all pancreatic surgery-certified centres in the Netherlands, which already have a long-standing collaboration^16^. This does not guarantee that the implementation strategy with regard to the oncological treatment was fully executed in non-DPCG centres in the Netherlands, as infrastructures in healthcare regions built around DPCG centres vary in care logistics and execution of referrals^58^. The predefined surgical safety benchmarks were established based on the in-hospital or 30-day postoperative interval, which may have resulted in missed additional major morbidity^18^. Nevertheless, the 90-day mortality rate of 3% including a 1% rate related to complications suggests a limited clinically relevant amount of additional severe morbidity. Lastly, data collection was performed by a single experienced clinical researcher and medical doctor who were not independent. The major strength of this study is that it concerns the first prospective trial that implemented and standardized the oncological and surgical care of patients with LAPC at a nationwide level. This approach of building a training programme with a subsequent implementation structure supported by external experts could be used for the improvement of surgical care in other areas, although this may require adjustments based on the healthcare system and regional care networks. When interpreting the results from the PREOPANC-4 implementation trial, it is crucial to realize that the implementation was executed in pancreas surgery-certified centres between which there has been long-standing collaboration (currently >15 years)^59^. Expansion of the indications for LAPC surgery requires pre-existent multidisciplinary experience in the care of patients with LAPC. In the near future, the PREOPANC-4 approach will be further distributed by a European international variant of the PREOPANC-4 implementation trial (PALACROS trial), led by the DPCG and several European LAPC expert centres.

## Supplementary Material

znag075_Supplementary_Data

## Data Availability

The data are not publicly available, but will be shared on reasonable request to the corresponding author.

## Collaborators

Adriënne te Riele (St. Jans Gasthuis, Weert, The Netherlands); Aniek Vlijm (Amsterdam UMC, location University of Amsterdam, Amsterdam, The Netherlands); Ankie van der Velden (Tergooi MC, Hilversum, The Netherlands); Anne M. E. Bruynzeel (Amsterdam UMC, location Vrije University, Amsterdam, The Netherlands); Anne M. Stiggelbout (Leiden University Medical Center, Leiden, The Netherlands); Anne-Marie Dietvorst (Het Van Weel-Bethesda Ziekenhuis, Dirksland, The Netherlands); Annemarie S. B. Conijn-Mensink (Zaans Medisch Centrum, Zaandam, The Netherlands); Annebeth Haringhuizen (Ziekenhuis Gelderse Vallei, Ede, The Netherlands); Anneke Roeterdink (Amsterdam UMC, location University of Amsterdam, Amsterdam, The Netherlands); Annette van Zweeden (Amstelland Ziekenhuis, Amstelveen, The Netherlands); Anouk van Asseldonk (Jeroen Bosch Hospital, ‘s Hertogenbosch, The Netherlands); Ammar A. Javed (NYU Langone Health, New York, NY, United States); André Sterk (Amsterdam UMC, location University of Amsterdam, Amsterdam, The Netherlands); Annuska Schoorlemmer (Amsterdam UMC, location University of Amsterdam, Amsterdam, The Netherlands); Anja Stam (Medisch Spectrum Twente, Enschede, The Netherlands); Aram van Brussel (Beatrix Ziekenhuis, Gorinchem, The Netherlands); Barbara M. Zonderhuis (Amsterdam UMC, location Vrije University, Amsterdam, The Netherlands); Bas Boekestijn (Leiden University Medical Center, Leiden, The Netherlands); Bibi Martens (Maastricht University Medical Center, Maastricht, The Netherlands); Brigitte Haberkorn (Maasstad Ziekenhuis, Rotterdam, The Netherlands); C. Yung Nio (Amsterdam UMC, location University of Amsterdam, Amsterdam, The Netherlands); Celine Leenen (Noordwest Ziekenhuisgroep, Alkmaar, The Netherlands); Daud Allajar (St. Jansdal Ziekenhuis, Harderwijk, The Netherlands); Deborah Reijnders-Tromp (St. Jans Gasthuis, Weert, The Netherlands); Dirk-Jan de Groot (University Medical Center Groningen, University of Groningen, Groningen, The Netherlands); Els Wink-Van Gestel (Meander Medisch Centrum, Amersfoort, The Netherlands); Emile D. Kerver (OLVG, Amsterdam, The Netherlands); Eran van Veldhuisen (Amsterdam UMC, location University of Amsterdam, Amsterdam, The Netherlands); Eva Versteijne (Amsterdam UMC, location University of Amsterdam, Amsterdam, The Netherlands); Evelien van Alphen (Amsterdam UMC, location University of Amsterdam, Amsterdam, The Netherlands); Evert van den Broek (University of Groningen and University Medical Center Groningen, Groningen, The Netherlands); Geert A. Cirkel (Regional Academic Cancer Center Utrecht, University Medical Center Utrecht/St. Antonius Hospital Nieuwegein, The Netherlands); Geertjan van Tienhoven (Amsterdam UMC, location University of Amsterdam, Amsterdam, The Netherlands); Geeske Heringa (IJsselland Ziekenhuis, Capelle aan den IJssel, The Netherlands); Gert Stockmans (Ziekenhuis Oost-Limburg, Genk, Belgium); Gian Piero Serafino (Jeroen Bosch Hospital, ‘s Hertogenbosch, The Netherlands); Hannàh N. Lelieveld-Rier (Amphia Hospital, Breda, The Netherlands); Hanneke W. M. van Laarhoven (Amsterdam UMC, location University of Amsterdam, Amsterdam, The Netherlands); Hanneke Zuetenhorst (Franciscus Gasthuis, Rotterdam, The Netherlands); Haske van Veenendaal (Erasmus University Rotterdam, Rotterdam, The Netherlands); Hetty van Mierlo (Meander Medisch Centrum, Amersfoort, The Netherlands); Ingeborg Griffioen (Leiden University Medical Center, Leiden, The Netherlands); Ingmar F. Rompen (Amsterdam UMC, location University of Amsterdam, Amsterdam, The Netherlands); Irene E. G. van Hellemond (Catharina Hospital, Eindhoven, The Netherlands); Iryna Symarska (Maastricht University Medical Center, Maastricht, The Netherlands); Jacques Peters (Radboud University Medical Center, Nijmegen, The Netherlands); Jan-Willem B. de Groot Isala, Zwolle, The Netherlands.); Jeanette Ham (Amstelland Ziekenhuis, Amstelveen, The Netherlands); Jeroen Willems (Anna Ziekenhuis, Geldrop, The Netherlands); Jessica Coes (Ziekenhuisgroep Twente, Almelo & Hengelo, The Netherlands); Joachim Kikomeko (Spaarne Gasthuis, Haarlem, The Netherlands); Johan van Rooijen (Martini Ziekenhuis, Groningen, The Netherlands); Joanne Verheij (Amsterdam UMC, location University of Amsterdam, Amsterdam, The Netherlands); Joost Nederend (Catharina Hospital, Eindhoven, The Netherlands); Mirte Streppel (Amphia Hospital, Breda, The Netherlands); José Schellekens (Bravis Ziekenhuis, Bergen op Zoom & Roosendaal, The Netherlands); Kishan R. D. Lutchman (Amsterdam UMC, location University of Amsterdam, Amsterdam, The Netherlands); Judith de Vos-Geelen (Maastricht University Medical Center, Maastricht, The Netherlands); Laura R. Moolenaar (Amsterdam UMC, VU University Medical Centre, Amsterdam, The Netherlands); Laurens van der Waaij (Martini Ziekenhuis, Groningen, The Netherlands); Laurens Beerepoot (Elisabeth-TweeSteden Ziekenhuis, Tilburg, The Netherlands); Leonie J. Mekenkamp (Medisch Spectrum Twente, Enschede, The Netherlands); Lilly H. J. Brada (Amsterdam UMC, location University of Amsterdam, Amsterdam, The Netherlands); Lobke van Leeuwen—Snoeks (Diakonessenhuis, Utrecht, The Netherlands); Lodewijk Brosens (Radboud University Medical Center, Nijmegen, The Netherlands); Linda Garms (Radboud University Medical Center, Nijmegen, The Netherlands); Leonieke W. Kranenburg (Erasmus University Medical Center, Rotterdam, The Netherlands); Loes J. Peters (Amsterdam UMC, location University of Amsterdam, Amsterdam, The Netherlands); Maartje Los (Regional Academic Cancer Center Utrecht, University Medical Center Utrecht/St. Antonius Hospital Nieuwegein, The Netherlands); Marco B. Polée (Medical Center Leeuwarden, Leeuwarden, The Netherlands); Marjolein Y. V. Homs (Erasmus MC Cancer Institute, University Medical Center, Rotterdam, The Netherlands); Marit Suttorp (Het Groene Hart Ziekenhuis, Gouda, The Netherlands); Marieke S. Walma (Regional Academic Cancer Center Utrecht, University Medical Center Utrecht / St. Antonius Hospital Nieuwegein, Nieuwegein & Utrecht, The Netherlands); Marjon Oostdijk (Admiraal de Ruyterziekenhuis, Goes, The Netherlands); Marleen Duizer (Streekziekenhuis Koningin Beatrix Winterswijk, Winterswijk, The Netherlands); Melanie F. M. A. Marting (Maastricht University Medical Center, Maastricht, The Netherlands); Michael Doukas (Erasmus MC Cancer Institute, University Medical Center, Rotterdam, The Netherlands); Miriam L. Wumkes (Jeroen Bosch Hospital, ‘s Hertogenbosch, The Netherlands); Monica Seijbel (Erasmus MC Cancer Institute, University Medical Center, Rotterdam, The Netherlands); Nicole D. Hildebrand (Maastricht University Medical Center, Maastricht, The Netherlands); Nynke M. Michiels (Leiden University Medical Center, Leiden, The Netherlands); Olaf Loosveld (Amphia Hospital, Breda, The Netherlands); Peter Nieboer (Wilhelmina Ziekenhuis Assen, Assen, The Netherlands); Ramon Bax (Catharina Hospital, Eindhoven, The Netherlands); Roel Haen (Erasmus MC Cancer Institute, University Medical Center, Rotterdam, The Netherlands); Roel Heesakkers (Jeroen Bosch Hospital, ‘s , Hertogenbosch, The Netherlands); Ronald M. van Dam (Maastricht University Medical Center, Maastricht, The Netherlands); Rosa van der Werf (Leiden University Medical Center, Leiden, The Netherlands); Rowan van Gerven (Albert Schweitzer Ziekenhuis, Dordrecht, The Netherlands); Rune van de Wetering (Slingeland Ziekenhuis, Doetinchem, The Netherlands); Rutger T. Theijse (Amsterdam UMC, location University of Amsterdam, Amsterdam, The Netherlands); Ruud Blankenburgh (Saxenburgh Medisch Centrum, Hardenberg, The Netherlands); Sandra Bakker (Zaans Medisch Centrum, Zaandam, The Netherlands); Sanne Achten (Catharina Hospital, Eindhoven, The Netherlands); Saskia A.C. Luelmo (Leiden University Medical Center, Leiden, The Netherlands); Simone Augustinus (Amsterdam UMC, location University of Amsterdam, Amsterdam, The Netherlands); Simone Rötgerink (OLVG, Amsterdam, The Netherlands); Stijn Crobach (Leiden University Medical Center, Leiden, The Netherlands); K.C. Khe Tran (Erasmus MC Cancer Institute, University Medical Center, Rotterdam, The Netherlands); Tess M.E. van Ramshorst (Rode Kruis Ziekenhuis, Beverwijk, The Netherlands); Wendy van der Deure-Gielisse (Het Groene Hart Ziekenhuis, Gouda, The Netherlands); Wouter W. te Riele (Regional Academic Cancer Center Utrecht, University Medical Center Utrecht/St. Antonius Hospital Nieuwegein, Utrecht & Nieuwegein, The Netherlands).
