# Supplementary material for: Surgical outcomes from nationwide implementation of the international best practice for locally advanced pancreatic cancer (PREOPANC-4) study
Source: Br J Surg. 2026 Jun 22;113(9):znag075. doi: 10.1093/bjs/znag075 (PMC13541110; doi:10.1093/bjs/znag075)
Supplement: Supplementary file 1 [file znag075_supplementary_data.docx]

**Surgical Outcomes from Nationwide Implementation of the International Best-Practice for Locally Advanced Pancreatic Cancer (PREOPANC-4) study**

Thomas F. Stoop, MD, PhD^1-3^*; Leonard W.F. Seelen, MD^4^*; Freek R. van ‘t Land, MD, PhD^5^*; Jacobien C.M. Scheepens, MD^4^; Mahsoem Ali, BSc^2,6^; Anna C. van der Hout, MA^7^; B. Marion van der Kolk, MD, PhD^8^; Bert A. Bonsing, MD^9^; Daan J. Lips, MD, PhD^10^; Eric R. Manusama, MD, PhD^11^; François E.J.A. Willemsen, MD, PhD^12^; Freek Daams, MD, PhD^2,6^; Geert Kazemier, MD, PhD^2,6^; Gijs A. Patijn, MD, PhD^13^; Ignace H. de Hingh, MD, PhD^14^; Jan H. Wijsman, MD, PhD^15^; Jennifer Schreinemakers, MD, PhD^15^; Joris I. Erdmann, MD, PhD^1,2^; J. Sven D. Mieog, MD, PhD^9^; Joost M. Klaase, MD, PhD^16^; Judith A.C. Rietjens, MSc, PhD^7,17^; Koop Bosscha, MD, PhD^18^; Lysanne P.M. Beuk, MD^5^; Maarten W. Nijkamp, MD, PhD^16^; Marcel den Dulk, MD, PhD^19,20^; Marnix P.M. Kop, MD^2,21^; Mike S.L. Liem, MD, PhD^10^; Misha Luyer, MD, PhD^14^; Martijn W.J. Stommel, MD, PhD^8^; Olivier R. Busch, MD, PhD^1,2^; Sebastiaan Festen, MD, PhD^22^; Stefan Bouwense, MD, PhD^19,20^; Tom M. Karsten, MD, PhD^22^; Tom W. van Ravens, MD^9^; Ulff P. Neumann, MD, PhD^20,23^; Vincent E. de Meijer, MD, PhD^16^; Vincent B. Nieuwenhuijs, MD, PhD^13^; Werner A. Draaisma, MD, PhD^18^; Wouter Derksen, MD, PhD^4^; Thomas L. Bollen, MD, PhD^24^; Bas Groot Koerkamp, MD, PhD^5^; Casper H.J. van Eijck, MD, PhD^5^; I. Quintus Molenaar, MD, PhD^4^; Christopher L. Wolfgang, MD, PhD, FACS^25^; Marco Del Chiaro, MD, PhD, FACS^3^; Matthew H.G. Katz, MD, PhD, FACS^26^; Thilo Hackert, MD, PhD^27^; Johanna W. Wilmink, MD, PhD^2,28#^; Hjalmar C. van Santvoort, MD, PhD^4#^; Roeland F. de Wilde, MD, PhD^5#^; Marc G. Besselink, MD, MSc, PhD^1,2#^ for the Dutch Pancreatic Cancer Group

^1^Amsterdam UMC, location University of Amsterdam, Department of Surgery, Amsterdam, the Netherlands; ^2^Cancer Center Amsterdam, Amsterdam, the Netherlands; ^3^Division of Surgical Oncology, Department of Surgery, University of Colorado Anschutz Medical Campus, Aurora, Colorado, United States; ^4^Department of Surgery, Regional Academic Cancer Center Utrecht, University Medical Center Utrecht / St. Antonius Hospital Nieuwegein, the Netherlands; ^5^Department of Surgery and Pulmonology, Erasmus MC Cancer Institute, University Medical Center, Rotterdam, the Netherlands; ^6^Amsterdam UMC, location Vrije Universiteit, Department of Surgery, Amsterdam, the Netherlands; ^7^Department of Public Health, Erasmus MC, University Medical Center Rotterdam, Rotterdam, the Netherlands; ^8^Department of Surgery, Radboud University Medical Center, Nijmegen, the Netherlands; ^9^Department of Surgery, Leiden University Medical Center, Leiden, the Netherlands; ^10^Department of Surgery, Medisch Spectrum Twente, Enschede, the Netherlands; ^11^Department of Surgery, Medical Center Leeuwarden, Leeuwarden, the Netherlands; ^12^Department of Radiology; Erasmus MC, University Medical Center Rotterdam, Rotterdam, the Netherlands; ^13^Department of Surgery, Isala, Zwolle, the Netherlands; ^14^Department of Surgery, Catharina Hospital, Eindhoven, the Netherlands; ^15^Department of Surgery, Amphia Ziekenhuis, Breda, the Netherlands; ^16^Department of Surgery, University of Groningen and University Medical Center Groningen, Groningen, the Netherlands; ^17^Department of Design, Organisation and Strategy, Faculty of Industrial Design Engineering, Delft University of Technology, Delft, the Netherlands; ^18^Department of Surgery, Jeroen Bosch Hospital, ‘s Hertogenbosch, the Netherlands; ^19^Department of Surgery, Maastricht University Medical Center, Maastricht, the Netherlands; ^20^Nutrim School for Nutrition and Translational Research in Metabolism, Maastricht, the Netherlands; ^21^Amsterdam UMC, location University of Amsterdam, Amsterdam, the Netherlands; ^22^Department of Surgery, OLVG, Amsterdam, the Netherlands; ^23^Department of Surgery, University Hospital Essen, Essen, Germany; ^24^Department of Radiology, St. Antonius Hospital, Nieuwegein, the Netherlands; ^25^Department of Surgery, NYU Langone Health, New York, New York, United States; ^26^Department of Surgical Oncology, The University of Texas MD Anderson Cancer Center, Houston, Texas, United States; ^27^Department of General, Visceral and Thoracic Surgery, University Hospital Hamburg-Eppendorf, Hamburg, Germany; ^28^Amsterdam UMC, location University of Amsterdam, Department of Medical Oncology, Amsterdam, the Netherlands;

** shared first author; # shared senior authorship*

**COLLABORATORS**

Adriënne te Riele; Aniek Vlijm; Ankie van der Velden; Anne Bruynzeel; Anne M. Stiggelbout; Anne-Marie Dietvorst; Annemarie S.B. Conijn-Mensink; Annebeth Haringhuizen; Anneke Roeterdink; Annette van Zweeden; Anouk van Asseldonk; Ammar A. Javed; André Sterk; Annuska Schoorlemmer; Anja Stam; Aram van Brussel; Barbara M. Zonderhuis; Bas Boekestijn; Bibi Martens; Brigitte Haberkorn; C. Yung Nio; Celine Leenen; Daud Allajar; Deborah Reijnders-Tromp; Dirk-Jan de Groot; Els Wink-Van Gestel; Emile D. Kerver; Eran van Veldhuisen; Eva Versteijne; Evelien van Alphen; Evert van den Broek; Geert A. Cirkel; Geertjan van Tienhoven; Geeske Heringa; Gert Stockmans; Gian Piero Serafino; Hannàh N. Lelieveld-Rier; Hanneke W.M. van Laarhoven; Hanneke Zuetenhorst; Haske van Veenendaal; Hetty van Mierlo; Ingeborg Griffioen; Ingmar F. Rompen; Irene E.G. van Hellemond; Iryna Symarska; Jacques Peters; Jan-Willem B. de Groot; Jeanette Ham; Jeroen Willems; Jessica Coes; Joachim Kikomeko; Johan van Rooijen; Joanne Verheij; Joost Nederend; Mirte Streppel; José Schellekens; Kishan R.D. Lutchman; Judith de Vos-Geelen; Laura Moolenaar; Laurens van der Waaij; Laurens Beerepoot; Leonie J. Mekenkamp; Lilly H.J. Brada; Lobke van Leeuwen – Snoeks; Lodewijk Brosens; Linda Garms; Leonieke W. Kranenburg; Loes J. Peters; Maartje Los; Marco B. Polée; Marjolein Y.V. Homs; Marit Suttorp; Marieke S. Walma; Marjon Oostdijk; Marleen Duizer; Melanie F.M.A. Marting; Michael Doukas; Miriam L. Wumkes; Monica Seijbel; Nicole D. Hildebrand; Nynke M. Michiels; Olaf Loosveld; Peter Nieboer; Ramon Bax; Roel Haen; Roel Heesakkers; Ronald M. van Dam; Rosa van der Werf; Rowan van Gerven; Rune van de Wetering; Rutger T. Theijse; Ruud Blankenburgh; Sandra Bakker; Sanne Achten; Saskia A.C. Luelmo; Simone Augustinus; Simone Rötgerink; Stijn Crobach; K.C. Khe Tran; Tess M.E. van Ramshorst; Wendy van der Deure-Gielisse; Wouter W. te Riele

**Corresponding author**

**Thomas F. Stoop, MD, PhD**

Department of Surgery, Amsterdam UMC

University of Amsterdam, Cancer Center Amsterdam

De Boelelaan 1117 (ZH-7F), 1081 HV Amsterdam, the Netherlands

t.f.stoop@amsterdamUMC.nl

**Marc G. Besselink, MD, MSc, PhD**

Department of Surgery, Amsterdam UMC

University of Amsterdam, Cancer Center Amsterdam

De Boelelaan 1117 (ZH-7F), 1081 HV Amsterdam, the Netherlands

[m.g.besselink@amsterdamUMC.nl](mailto:m.g.besselink@amsterdamUMC.nl)

**X**: @TFStoop, @chiaro_del & @MarcBesselink @BasGrootKoerkam @Caspervaneijck @cwolfga@

**BlueSky**: @TFStoop.bsky.social @MarcBesselink.bsky.social @BasGrootKoerkamp.bsky.social

**Supplementary Materials - Index**

| **Supplementary Methods** |  |
| --- | --- |
| **Appendix 1.** Training program and surgical principles | *page 4* |
| **Appendix 2.** Resectability criteria for locally advanced pancreatic cancer | *page 8* |
| **Appendix 3.** Clinical protocol | *page 9* |
| **Appendix 4.** Work-up for patients with postoperative diarrhoea | *page 10* |
| **Supplementary Figures and Tables** |  |
| **Appendix 5**. Vascular involvement at diagnosis | *page 11* |
| **Appendix 6**. Surgical details | *page 12* |
| **Appendix 7**. Reoperation | *page 15* |
| **Appendix 8**. Surgical outcome stratified for resectability definition | *page 17* |
| **Appendix 9**. Surgical details and outcome per type of extended resection | *page 19* |
| **Appendix 10**. Surgical details and outcome per type of pancreatic resection | *page 20* |
| **References** | *page 21* |
|  |  |

**Supplementary Methods**

**APPENDIX 1**. Training program and surgical principles ^1^

- **Training program**

Participants in the training program were the multidisciplinary teams from all seven Dutch university medical centres. The three highest volume centres in pancreatic surgery including LAPC surgery (i.e., Amsterdam UMC, ErasmusMC, RACU) functioned as referral centres for LAPC surgery during the implementation.^2^ The four other university centres (i.e., UMCG, MUMC+, LUMC, Radboud UMC) participated in the training program, since the Dutch Pancreatic Cancer Group (DPCG) is working towards a situation in which LAPC surgery is concentrated in five centres in the Netherlands.

The training program comprised a theoretical and practical training program, organized by the four international experts. The training program included three steps: (1) multidisciplinary training program; (2) surgical technique training and off-site proctoring; and (3) on-site proctoring.

*Step 1 – Multidisciplinary training program:* The proctors educated the Dutch multidisciplinary teams from the selected Dutch centres on induction therapy regimens and patient selection; response evaluation; indications for surgery, based on scientific evidence and expert opinion. The proctors organized additional webinars for the pancreatic surgeons to discuss intraoperative decision-making and surgical techniques.

*Step 2 – Surgical technique training and off-site proctoring:* The proctors organized a training session in the Skills Lab & Simulation Center form the ErasmusMC for the pancreatic surgeons from the seven university medical centers. This session consisted of lectures and practical skills training on the approach of peripancreatic vasculature; (contra-)indications for and techniques of arterial divestment; pitfalls and (contra-)indications for portomesenteric venous and arterial resections (and reconstructions); and perioperative monitoring and anti-coagulants regimens. Thereafter, the pancreatic surgeons from each of the three high-volume Dutch centres were surgically trained via off-site proctoring during several live LAPC surgeries in the proctors’ centres.

*Step 3 – On-site proctoring:* The pancreatic surgeons from the three highest-volume Dutch centres were proctored in their own institution by the proctor(s) during LAPC surgery. Pancreatic surgeons from the four observing university medical centres joined these sessions.

**- Surgical principles -**

Staging laparoscopy was recommended at the start of surgery to exclude occult metastases and intraoperative ultrasonography (IOUS) could be considered to assess the extent of vascular involvement.^3,4^ In the absence of metastatic disease, an artery-first approach was recommended to assess the arterial involvement first and obtain control over the involved vasculature.^5^ A mesocaval shunt or meso-portal bypass at the start of the procedure was considered in case of extensive collaterals due to venous occlusion by thrombus or tumour involvement. In this way, the portal hypertension through the venous collaterals could be released, minimizing the risk of major bleeding during the dissection phase. Once the specimen was removed, the portomesenteric continuity could be restored by end-to-end reconstruction or shortening the graft that was used for the shunt or bypass.^6^

Periadventitial arterial divestment in patients with arterial involvement was performed if there was no evidence of arterial wall ingrowth on preoperative cross-sectional imaging and intraoperatively (i.e., no string sign, negative frozen biopsies, peri-arterial tissue can be peeled off the artery without significant resistance).^7,8^ Importantly, tumour in-growth in the superior mesenteric artery (SMA) on preoperative cross-sectional imaging (i.e., string sign) requiring an SMA resection to achieve a radical resection was considered to be a contraindication for surgery,^8^ given the associated high short-term mortality of 7% in even very high-volume expert centres.^9-11^

In patients requiring left-sided pancreatectomy with coeliac axis resection, there was no routine use of preoperative embolization of the common hepatic artery (CHA) and no routine arterial reconstruction when the proper hepatic artery (PHA) and gastroduodenal artery (GDA) were not involved.^12^ Generally, tumours involving the PHA, right hepatic artery (RHA), and/or left hepatic artery (LHA) were only operated in highly selected patients, depending on the vessel’s calibre and presence of a distal landing zone for reconstruction, even in absence of a string sign on cross-sectional imaging given the risk for iatrogenic damage during divestment.^8^

If an arterial reconstruction was performed, total pancreatectomy was not routinely performed but rather based on the surgeon’s discretion, taking into account risk factors for postoperative pancreatic fistula (POPF) and the location of the arterial anastomosis.^13^ After all, in these locally advanced tumours, the pancreas parenchyma is often fibrotic with a dilated pancreatic duct in case of a pancreatic head tumour, resulting in a low risk of clinically relevant POPF is low and erosive postpancreatectomy hemorrhage (PPH).^14^

In case of portomesenteric venous involvement requiring a segmental PVR, the need for an interposition graft was reduced by a Cattell-Braasch maneuver, allowing complete mobilization of the intestines and mesentery.^15^ Other methods to enhance the length for an end-to-end portomesenteric reconstruction was mobilization of the liver, splenic vein transection, and portal vein dissection towards its hilar bifurcation. Nevertheless, if an interposition graft is still needed to create a tension-free anastomosis, an autologous graft, tubularized Bovine patch, or cadaveric graft was preferred over alloplastic grafts considering the increased risk of portomesenteric venous thrombosis.^16^ The use of specific intra- and postoperative anti-coagulant strategies was not standardized given the lack of strong evidence.^17^

| **APPENDIX 2.** DPCG criteria for locally advanced pancreatic cancer | | | | | |
| --- | --- | --- | --- | --- | --- |
|  | **SMA** | **(C)HA** | **CA** | **SMV/PV** |  |
| **DPCG definition** | >90° | >90° | >90° | >270° or occlusion |  |
| **NCCN definition** |  |  |  |  |  |
| *Pancreatic head/*  *uncinate process* | >180° | - | >180° | Unreconstructible due to tumour involvement or occlusion |  |
| *Pancreatic body/tail* | >180° | CHA involvement with extension to CA or hepatic artery bifurcation | >180° | Unreconstructible due to tumour involvement or occlusion |  |
| *DPCG,* Dutch Pancreatic Cancer Group; *NCCN,* National Comprehensive Cancer Network; *SMA,* superior mesenteric artery; *(C)HA,* (common) hepatic artery; *CA,* coeliac axis; *SMV,* superior mesenteric vein; *PV,* portal vein. | | | | | |

**APPENDIX 3**. Clinical protocol

The following modifications concerning patient selection were made in the clinical protocol during the implementation phase. First, the biological criterion at response evaluation for proceeding to surgery after chemotherapy was a relative reduction in serum CA19-9 of ≥30%.^18^ Ideally, serum CA19-9 was measured at time of non-elevated serum bilirubin. From July 2023, this criterion was further nuanced with an additional criterion that ideally the serum CA19-9 should be <500 U/ml following induction chemotherapy, based on evolving evidence illustrating the value of both the relative response as well as the absolute value of CA19-9 after induction chemotherapy.^19-21^ The following nuances were made for the interpretation of the relative and absolute CA19-9 levels after at least four months of chemotherapy:

- In case of CA19-9 reduction ≥30%, but serum CA19-9 >500 U/ml despite a still decreasing trend during the last cycles of chemotherapy, continuation of chemotherapy should be considered up to a total of six months, followed by restaging.
- In case of CA19-9 reduction ≥30%, but CA19-9 >500 U/ml and stabilized during the last cycles of chemotherapy without further decrease, surgery with the intention of resection should be considered.

Second, a protocol was designed that aimed to better diagnose and more adequately treat postoperative neurogenic diarrhoea. See **Appendix 4** for the work-up for patients with postoperative diarrhoea.^22,23^

Third, based on the 2023 European Society for Medical Oncology (ESMO) guideline, patients with a high suspicion for having pancreatic cancer but without pathological confirmation after multiple fine needle aspirations or biopsies were allowed for inclusion if the final histopathology after resection confirmed the diagnosis of pancreatic adenocarcinoma.^24^

**APPENDIX 4.** Step-up approach for patients with postoperative diarrhoea

**Non-opioid anti-diarrhoeal drugs**

*Add bulking agents (e.g., psyllium fibers [Metamucil], albumin tannate, natural aluminum silicate)* with stepwise dose escalation

**Opioid anti-diarrhoeal drugs**

*Add loperamide* with stepwise dose escalation*

**> Optimization of pancreatic enzymes**

**> Standard dose PPI**

**Exclusion of other causes** (e.g., Clostridium difficile enteritis, bacterial overgrowth [hydrogen breathing test], dumping syndrome, exocrine insufficiency)

**> PPI** stepwise dose escalation

**> Consultation of dietitian**

**Opioid anti-diarrhoeal drugs**

*Add opium tincture* with stepwise dose escalation

**Octreotide** (long acting)

Start with test dose for adverse events

*Eventually adding codeine with stepwise. This step-up approach is published in BJS Open.^24^

**Supplementary Figures and Tables**

| **APPENDIX 5.** Vascular involvement at diagnosis | |  |  |
| --- | --- | --- | --- |
| **Variables** | **Resections for LAPC** | |  |
| Portomesenteric venous involvement, *n* (%)  None  ≤ 180° | 16 (10)  80 (52) | |  |
| > 180° | 58 (38) | |  |
| *No lumen reduction*  *Yes, ≤50% narrowing*  *Yes, >50% narrowing*  *Yes, occlussion*  *Missing*  Length of involvement (median, IQR) (mm)  *Missing*, *n* (%) | 36 (26)  35 (25)  46 (33)  18 (13)  3 (2)  24 (15 to 30)  2 (1) | |  |
| Superior mesenteric artery involvement, *n* (%)  None  ≤ 180°  > 180° | 51 (33)  75 (49)  28 (18) | |  |
| Coeliac axis involvement, *n* (%)  None  ≤ 180°  > 180°  Any hepatic artery, *n* (%) | 115 (75)  16 (10)  23 (15) | |  |
| None  ≤ 180°  > 180° | 73 (47)  40 (26)  41 (27) | |  |
| *n,* number of patients | | | |

| **APPENDIX 6.** Surgical details | |
| --- | --- |
| **Variables** | **Resections for LAPC** |
| **Portomesenteric venous resection** | ***n* = 92** |
| Reconstruction material  PVR ISGPS type 2  *Autologous vein*  *Falciform ligament*  *Bovine patch*  PVR ISGPS type 4  *Autologous vein*  *Falciform ligament*  *Alogenous graft*  *Bovine patch*  *Synthetic prosthesis* | 7 (8)  1 (14)  4 (57)  2 (29)  9 (10)  1 (11)  2 (22)  1 (11)  3 (33)  2 (22) |
| Length of resection (median, IQR) (mm) ^#^  PVR ISGPS type 1 (missing *n*=2)  PVR ISGPS type 2  PVR ISGPS type 3 (missing *n*=3)  PVR ISGPS type 4 | 30 (20 to 40)  15 (9 to 20)  35 (30 to 43)  30 (25 to 40)  40 (30 to 48) |
| Mesocaval shunt before resection phase, *n* (%) | 1 (1) |
| Porto-mesenteric bypass before resection phase, *n* (%) | 1 (1) |
| Splenic vein transection, *n* (%) | 37 (40) |
| Ligation of jejunal / ileal branches, *n* (%)  Missing, *n* (%) | 40 (44)  2 (2) |
| Portomesenteric venous clamping, *n* (%) * | 90 (98) |
| Clamping time (median, IQR) (min) ** | 16 (12 to 20) |
| Superior mesenteric artery clamping, *n* (%) | 7 (8) |
| Clamping time (median, IQR) (min) | 15 (6 to 45) |
| Bowel congestion, *n* (%)  No / temporary without clinical consequences  Yes, congestion / malperfusion requiring enteral resection  Missing | 82 (89)  3 (3)  7 (8) |
| Type of surgeon for vascular reconstruction, *n* (%) |  |
| HPB surgeon  Surgeon who is both HPB + transplant surgeon  HPB surgeon with previous transplant experience  Transplant surgeon  Vascular surgeon | 39 (42)  45 (49)  18 (20)  3 (3)  4 (4) |
| **Arterial divestment** | ***n* = 119** |
| Superior mesenteric artery, *n* (%)  ≤ 180°  > 180°  Missing | 100 (84)  24 (24)  40 (40)  36 (36) |
| Hepatic artery, *n* (%)  ≤ 180°  > 180°  Missing | 53 (34)  3 (6)  24 (45)  26 (49) |
| Coeliac axis, *n* (%) | 22 (14) |
| **Hepatic artery resection** | ***n* = 19** |
| Indication, *n* (%)  Oncological indication (i.e., suspected tumour involvement)  Iatrogenic damage – injury during divestment  Iatrogenic damage – injury other then during divestment | 16 (84)  2 (11)  1 (5) |
| Hepatic artery branch, *n* (%)  Common hepatic artery  Proper hepatic artery  Right hepatic artery  Left hepatic artery  Aberrant hepatic artery  Accessory hepatic artery | 8 (42)  4 (21)  2 (11)  3 (16)  10 (53)  2 (11) |
| Type of resection, *n* (%)  Primary closure (i.e., ligation)  Segmental resection with E-T-E  Segmental resection with interposition graft | 9 (47)  8 (42)  2 (11) |
| Type of surgeon, *n* (%) |  |
| HPB surgeon  Surgeon who is both HPB + transplant surgeon  HPB surgeon with previous transplant experience  Transplant surgeon  Vascular surgeon | 8 (42)  7 (37)  4 (21)  1 (5)  4 (3) |
| **Coeliac axis resection** | ***n* = 14** |
| Preoperative embolization, *n* (%) | 3 (21) |
| Indication, *n* (%)  Oncological indication (i.e., suspected tumour involvement)  Iatrogenic damage – injury during divestment  Iatrogenic damage – injury other then during divestment | 14 (100)  0 (0)  0 (0) |
| Type of coeliac axis resection, *n* (%)  Without reconstruction  With reconstruction | 13 (93)  1 (7) |
| Gastric congestion, *n* (%)  No  Yes, transient venous gastric congestion  Yes, persistent venous gastric congestion  Missing | 10 (71)  2 (14)  1 (7)  1 (7) |
| Type of surgeon, *n* (%) |  |
| HPB surgeon  Surgeon who is both HPB + transplant surgeon  HPB surgeon with previous transplant experience  Transplant  Vascular surgeon | 7 (50)  4 (29)  3 (21)  1 (7)  2 (1) |
| **Superior mesenteric artery resection** | ***n* = 2** |
| Indication, *n* (%)  Oncological indication (i.e., suspected tumour involvement)  Iatrogenic damage – injury during divestment  Iatrogenic damage – injury other then during divestment | 0 (0)  2 (100)  0 (0) |
| Type of resection, *n* (%)  Wedge resection with Bovine patch reconstruction  Segmental resection with E-T-E | 1 (50)  1 (50) |
| SMA clamping time (min) | 45 & 55 |
| Bowel hypoxia, *n* (%)  No / yes, temporary without clinical consequences  Yes, ischemia with need for enteral resection | 2 (100)  0 (0) |
| Type of surgeon, *n* (%) |  |
| HPB surgeon  Surgeon who is both HPB + transplant surgeon  HPB surgeon with previous transplant experience  Vascular surgeon | 0 (0)  0 (0)  2 (100)  2 (100) |
| *n*, number of patients; #missing *n*=7; *missing *n*=1; **missing *n*=6 | |

| **APPENDIX 7.** Reoperation | | |
| --- | --- | --- |
| **Case** | **Primary operation** | **Indication for reoperation** |
| **#1** | PD + PVR ISGPS type 3 | Migrated Hot AXIOS stent from earlier endoscopic GJ, managed with removal of the stent and creation of a new GJ. |
| **#2** | PD + SMA divestment | Bile leak from a duct of Luschka, managed with closure. |
| **#3** | Left-sided pancreatic resection + PVR ISGPS 2, coeliac axis resection without reconstruction, SMA divestment and adrenalectomy.  NB1. Preoperatively embolization of the CHA was performed. Intraoperatively no gastric congestion. | Stomach perforation, managed with primary closure. Thereafter, a second relaparotomy was needed because of a stomach perforation again, managed with a sleeve gastrectomy. |
| **#4** | PD + PVR ISGPS type 3 and divestment of HA and SMA. | PPH from the pancreas nearby the PJ in absence of POPF as well as bleeding in the mesocolon, managed with ligation. |
| **#5** | Left-sided pancreatic resection + coeliac axis resection without reconstruction, adrenalectomy, and diaphragm resection. | Wound dehiscence including the fascia, managed with closure. |
| **#6** | PD + PVR ISGPS type 1 including multiple venous branches and collaterals + resection of mesocolon, and SMA divestment. Intraoperatively congestion of the intestines requiring an additional jejunal resection. | From surgery persistent hemodynamic instability with imminent multi-organ failure, wherefore diagnostic relaparotomy at postoperative day 1. Intraoperative extraction of fluids, without need for additional intestinal resection. |
| **#7** | PD + PVR ISGPS type 1 and CHA resection with end-to-end reconstruction (after using the splenic artery as jump graft). | Ischemia of pancreatic remnant, spleen, and distal stomach at postoperative day 1, managed with completion pancreatectomy combined with splenectomy and resection of the antrum. |
| **#8** | Left-sided pancreatic resection + PVR ISGPS type 3 and divestment of HA and SMA. | Iatrogenic gastric perforation made during drainage of intra-abdominal collections, managed with diagnostic laparoscopy with primary closure of the perforation. |
| **#9** | PD + SMA divestment and IVC resection. | SMA aneurysm and dissection with bleeding in false lumen, leading to bowel ischemia, managed endovascular stenting immediately followed by relaparotomy performing right hemicolectomy with ileostomy. |
| **#10** | PD + SMA divestment | Repositioning of luxated intra-abdominal drain, complicated by hemorrhage from the gastroduodenal artery wherefore coiling, complicated by liver ischemia from segment 2-3, managed with liver resection of segments 2-3. |
| **#11** | PD + SMA divestment. | Postoperative percutaneous transhepatic drain placement because of bile leak, complicated by hematothorax and liver bleeding, managed via a thoracotomy. |
| **#12** | Left-sided pancreatic resection + coeliac axis resection without reconstruction, PVR ISGPS type 4, and SMA divestment. | Relaparotomy because of necrotic gall bladder, managed with cholecystectomy. |
| **#13** | PD + PVR ISGPS type 3, HA and SMA divestment, and resection of mesocolon and additional jejunum. | Relaparotomy to remove a corpus alienum (i.e., surgical gaze left behind) |
| *PD,* pancreatoduodenectomy; *PVR,* portomesenteric venous resection; *ISGPS,* International Study Group for Pancreatic Surgery; *GJ,* gastrojejunostomosis; *SMA,* superior mesenteric artery; *HA,* hepatic artery; *PPH,* postpancreatectomy hemorrhage; *POPF,* postoperative pancreatic fistula; *IVC,* inferior vena cava; | | |

| **APPENDIX 8.** Surgical outcome after resection for LAPC stratified for resectability definition | | | |
| --- | --- | --- | --- |
| **Variables** | **NCCN LAPC** | **DPCG LAPC but not NCCN LAPC** |  |
| **Patients, *n*** | **57** | **97** |  |
| Type of centre, *n* (%)  Referral centre  Other university centre  Non-university centre | 46 (81)  9 (16)  2 (4) | 68 (70)  25 (26)  4 (4) |  |
| **INTRAOPERATIVE OUTCOME** |  |  |  |
| Intraoperative blood loss (median, IQR) (ml)  Missing, *n* (%) | 1100 (600 to 1800)  0 (0) | 800 (480 to 1500)  1 (1) |  |
| Intraoperative blood transfusion, *n* (%)  Number of transfusions (median, IQR) | 2 (1 to 3) | 2 (1 to 3) |  |
| Operation time (median, IQR) (min)  Missing, *n* (%) | 420 (340 to 526)  1 (2) | 405 (303 to 508)  1 (1) |  |
| Macrosopically radical resection (R0/R1) , *n* (%) | 56 (98) | 97 (100) |  |
| **POSTOPERATIVE OUTCOME** |  |  |  |
| Delayed gastric emptying, *n* (%)  Grade B  Grade C | 3 (5)  6 (11) | 8 (8)  9 (9) |  |
| Postpancreatectomy hemorrhage, *n* (%)  Grade B  Grade C | 10 (18)  1 (2) | 14 (14)  3 (3) |  |
| Postoperative pancreatic fistula, *n* (%)^#^  Grade B  Grade C | 10 (19)  0 (0) | 8 (9)  1 (1) |  |
| Bile leakage, *n* (%)^##^  Grade B  Grade C | 5 (13)  0 (0) | 3 (3)  1 (1) |  |
| Chyle leakage, *n* (%)  Grade B  Grade C | 6 (11)  0 (0) | 12 (12)  0 (0) |  |
| Major morbidity, *n* (%)  Reoperation, *n* (%)  Delayed reconstruction  Postoperative complication-related  Organ failure, *n* (%)  Single-organ failure  Multi-organ failure  MCU/ICU admission, *n* (%)  Length of stay (median, IQR) (days) | 28 (49)  9 (16)  2 (4)  7 (12)  1 (2)  0 (0)  1 (2)  5 (9)  4 (3 to 4) | 40 (41)  6 (6)  0 (0)  6 (6)  2 (2)  2 (2)  0 (0)  8 (8)  7 (5 to 8) |  |
| Hospital stay (median, IQR) (days) | 9 (6 to 15) | 9 (7 to 17) |  |
| In-hospital / 30-day mortality, *n* (%)  90-day mortality, *n* (%)  Complication-related  Disease-related  Other cause  Unknown | 1 (2)  3 (5)  1 (33)  0 (0)  1 (33)  1 (33) | 0 (0)  2 (2)  0 (0)  2 (100)  0 (0)  0 (0) |  |
| Readmission, *n* (%)^⌘^ | 17 (30) | 23 (24) |  |
| *n*, number of patients; *IQR,* interquartile range; *NCCN,* National Comprehensive Cancer Network; *LAPC,* locally advanced pancreatic cancer; *MCU*, medium care unit; *ICU*, intensive care unit; ⌘, patients who died during admission (i.e., in-hospital mortality) are excluded for the nominator; #, patients who underwent total pancreatectomy are excluded for the nominator; ##, patients who underwent left-sided pancreatectomy are excluded for the nominator. | | | |

| **APPENDIX 9**. Surgical details and outcome per type of extended resection | | | | |
| --- | --- | --- | --- | --- |
| **Variables** | **Standard pancreatic resection** | **Portomesenteric venous resection** | **Multivisceral resection** | **Arterial resection** |
|  | ***n* = 36** | ***n* = 57** | ***n* = 28** | ***n* = 32** |
| **Surgical procedure** | | | | |
| Type of pancreatic resection, *n* (%) |  |  |  |  |
| Pancreatoduodenectomy | 30 (83) | 49 (86) | 19 (68) | 14 (44) |
| Left pancreatic resection | 5 (14) | 3 (5) | 7 (25) | 14 (44) |
| Total pancreatectomy | 1 (3) | 5 (9) | 2 (7) | 4 (13) |
| Arterial divestment, *n* (%) | 30 (83) | 49 (86) | 23 (82) | 16 (50) |
| Superior mesenteric artery | 26 (72) | 38 (67) | 21 (75) | 14 (44) |
| Hepatic artery | 18 (50) | 22 (39) | 11 (39) | 2 (6) |
| Coeliac axis | 5 (14) | 6 (11) | 7 (25) | 4 (13) |
| Portomesenteric venous resection, *n* (%) | - | 57 (100) | 21 (75) | 14 (44) |
| PVR type 1-2 | - | 18 (32) | 4 (14) | 3 (9) |
| PVR type 3-4 | - | 36 (63) | 16 (57) | 11 (34) |
| No reconstruction | - | 4 (7) | 1 (4) | 0 (0) |
| Arterial resection, *n* (%) | - | - | - | 32 (100) |
| Superior mesenteric artery | - | - | - | 2 (6) |
| Hepatic artery | - | - | - | 19 (59) |
| Coeliac axis | - | - | - | 14 (44) |
| Multivisceral resection(s), *n* (%) | - | - | 28 (100) | 8 (25) |
| Stomach  (Meso)colon and/or its vasculature  Small bowel  Adrenal  Kidney and/or its vasculature  Diaphragm  Liver | -  -  -  -  -  -  - | -  -  -  -  -  -  - | 4 (14)  21 (75)  8 (29)  4 (14)  2 (7)  0 (0)  0 (0) | 1 (3)  2 (6)  1 (3)  5 (16)  0 (0)  2 (6)  0 (0) |
| **Surgical outcome** | | | | |
| Delayed gastric emptying, *n* (%)  Grade B  Grade C | 2 (6)  3 (8) | 4 (7)  5 (9) | 1 (4)  4 (14) | 4 (13)  3 (9) |
| Postpancreatectomy hemorrhage, *n* (%)  Grade B  Grade C | 3 (8)  2 (6) | 11 (19)  0 (0) | 5 (18)  1 (4) | 5 (16)  1 (3) |
| Postoperative pancreatic fistula, *n* (%)^#^  Grade B  Grade C | 5 (14)  1 (3) | 6 (12)  0 (0) | 3 (12)  0 (0) | 4 (13)  0 (0) |
| Bile leakage, *n* (%)^##^  Grade B  Grade C | 2 (6)  1 (3) | 4 (7)  0 (0) | 1 (5)  0 (0) | 1 (6)  0 (0) |
| Chyle leakage, *n* (%)  Grade B  Grade C | 4 (11)  0 (0) | 7 (12)  0 (0) | 2 (7)  0 (0) | 5 (16)  0 (0) |
| Major morbidity, *n* (%)  Reoperation, *n* (%)  Organ failure, *n* (%)  MCU/ICU admission, *n* (%) | 12 (33)  3 (8)  0 (0)  2 (6) | 24 (42)  3 (5)  0 (0)  3 (5) | 14 (50)  3 (11)  1 (4)  3 (11) | 17 (53)  5 (16)  1 (3)  4 (13) |
| Hospital stay (median, IQR) (days) | 8 (6 to 15) | 9 (6 to 16) | 11 (8 to 18) | 12 (7 to 20) |
| In-hospital / 30-day mortality, *n* (%)  90-day mortality, *n* (%) | 0 (0)  1 (3) | 0 (0)  1 (2) | 0 (0)  0 (0) | 1 (3)  3 (9) |
| Readmission, *n* (%)^⌘^ | 8 (22) | 14 (25) | 7 (25) | 10 (31) |
| *n*, number of patients; *IQR,* interquartile range; *MCU*, medium care unit; *ICU*, intensive care unit; ⌘, patients who died during admission (i.e., in-hospital mortality) are excluded for the nominator; #, patients who underwent total pancreatectomy are excluded for the nominator; ##, patients who underwent left-sided pancreatectomy are excluded for the nominator. | | | | |

| **APPENDIX 10**. Surgical details and outcome per type of pancreatic resection | | | | | | | | |  |
| --- | --- | --- | --- | --- | --- | --- | --- | --- | --- |
| **Variables** | **Pancreatoduodenectomy** | | **Left sided pancreatic resection** | | | **Total pancreatectomy** | | |  |
|  | **Standard**  ***n* = 30** | **Extended**  ***n* = 83** | **Standard**  ***n* = 5** | | **Extended**  ***n* = 24** | **Standard**  ***n* = 1** | **Extended**  ***n* = 11** |  |  |
| **Surgical procedure** | | | | | | | | |  |
| Arterial divestment, *n* (%) | 24 (80) | 69 (83) | 5 (100) | | 13 (54) | 1 (100) | 7 (64) |  |  |
| Superior mesenteric artery | 22 (73) | 59 (71) | 3 (60) | | 11 (46) | 1 (100) | 4 (36) |  |  |
| Hepatic artery | 12 (40) | 23 (28) | 5 (100) | | 6 (25) | 1 (100) | 6 (55) |  |  |
| Coeliac axis | 0 (0) | 8 (10) | 4 (80) | | 6 (25) | 1 (100) | 3 (27) |  |  |
| Portomesenteric venous resection, *n* (%) | - | 73 (88) | - | | 9 (38) | - | 10 (91) |  |  |
| PVR type 1-2 | - | 19 (23) | - | | 5 (21) | - | 1 (9) |  |  |
| PVR type 3-4 | - | 50 (60) | - | | 4 (17) | - | 9 (82) |  |  |
| No reconstruction | - | 5 (6) | - | | 0 (0) | - | 0 (0) |  |  |
| Arterial resection, *n* (%) | - | 14 (17) | - | | 14 (58) | - | 4 (36) |  |  |
| Superior mesenteric artery | - | 0 (0) | - | | 0 (0) | - | 2 (18) |  |  |
| Hepatic artery | - | 14 (17) | - | | 4 (17) | - | 1 (9) |  |  |
| Coeliac axis | - | 0 (0) | - | | 13 (54) | - | 1 (9) |  |  |
| Multivisceral resection(s), *n* (%)  Stomach  (Meso)colon and/or its vasculature  Small bowel  Adrenal  Kidney and/or its vasculature  Diaphragm  Liver | -  -  -  -  -  -  -  - | 21 (25)  0 (0)  19 (23)  6 (7)  0 (0)  1 (1)  0 (0)  0 (0) | -  -  -  -  -  -  -  - | | 11 (46)  2 (8)  3 (13)  2 (8)  8 (33)  1 (4)  2 (8)  0 (0) | -  -  -  -  -  -  -  - | 4 (36)  3 (27)  1 (9)  1 (9)  1 (9)  0 (0)  0 (0)  0 (0) |  |  |
| **Surgical outcome** | | | | | | | | |  |
| Delayed gastric emptying, *n* (%)  Grade B  Grade C | 2 (7)  3 (10) | 5 (6)  6 (7) | | 0 (0)  0 (0) | 2 (8)  4 (17) | 0 (0)  0 (0) | 2 (18)  2 (18) | | |
| Postpancreatectomy hemorrhage, *n* (%)  Grade B  Grade C | 3 (10)  2 (7) | 16 (19)  2 (2) | | 0 (0)  0 (0) | 4 (17)  0 (0) | 0 (0)  0 (0) | 1 (9)  0 (0) | | |
| Postoperative pancreatic fistula, *n* (%)  Grade B  Grade C | 5 (17)  1 (3) | 8 (10)  0 (0) | | 0 (0)  0 (0) | 5 (21)  0 (0) | -  - | -  - | | |
| Bile leakage, *n* (%)  Grade B  Grade C | 2 (7)  1 (3) | 5 (6)  0 (0) | | -  - | -  - | 0 (0)  0 (0) | 1 (9)  0 (0) | | |
| Chyle leakage, *n* (%)  Grade B  Grade C | 3 (10)  0 (0) | 10 (12)  0 (0) | | 0 (0)  0 (0) | 3 (13)  0 (0) | 1 (100)  0 (0) | 1 (9)  0 (0) | | |
| Major morbidity, *n* (%)  Reoperation, *n* (%)  Organ failure, *n* (%)  MCU/ICU admission, *n* (%) | 12 (40)  3 (10)  0 (0)  2 (7) | 35 (42)  7 (8)  1 (1)  6 (7) | | 0 (0)  0 (0)  0 (0)  0 (0) | 15 (63)  4 (17)  2 (8)  4 (17) | 0 (0)  0 (0)  0 (0)  0 (0) | 6 (55)  1 (9)  0 (0)  1 (9) | | |
| Hospital stay (median, IQR) (days) | 8 (7 to 15) | 10 (7 to 16) | | 5 (4 to 7) | 9 (6 to 20) | 8 (NE to NE) | 15 (12 to 19) | | |
| In-hospital / 30-day mortality, *n* (%)  90-day mortality, *n* (%) | 0 (0)  1 (3) | 0 (0)  3 (4) | | 0 (0)  0 (0) | 1 (4)  1 (4) | 0 (0)  0 (0) | 0 (0)  0 (0) | | |
| Readmission, *n* (%)^⌘^ | 8 (27) | 22 (27) | | 0 (0) | 7 (30) | 0 (0) | 3 (27) | | |
| *n*, number of patients; *IQR,* interquartile range; *MCU*, medium care unit; *ICU*, intensive care unit; *NE,* not estimable. ⌘, patients who died during admission (i.e., in-hospital mortality) are excluded for the nominator. | | | | | | | | |  |

**REFERENCES**

1.	Stoop TF, Seelen LWF, van ’t Land FR, et al. Nationwide Implementation of the International Multidisciplinary Best-Practice for Locally Advanced Pancreatic Cancer (PREOPANC-4): study protocol. *BMC Cancer*. 2025;25(1):299. doi:10.1186/s12885-025-13554-w

2.	Stoop TF, Seelen LWF, van ’t Land FR, et al. Nationwide use and outcome of surgery for locally advanced pancreatic cancer following induction chemotherapy. *Ann Surg Oncol.* 2024;31(4):2640-2653. doi:10.1245/s10434-023-14650-6

3.	Stoop TF, Lutchman KRD, Theijse RT, et al. Staging laparoscopy to detect occult metastases in localized pancreatic cancer: global survey among nine international societies. *HPB*. 2026;28(3):313-323. doi:10.1016/j.hpb.2025.12.001

4.	Michiels N, Doppenberg D, Groen JV, et al. Intraoperative Ultrasound During Surgical Exploration in Patients with Pancreatic Cancer and Vascular Involvement (ULTRAPANC): A Prospective Multicenter Study. *Ann Surg Oncol*. 2023;30(6):3455-3463. doi:10.1245/s10434-023-13112-3

5.	Muller PC, Muller BP, Hackert T. Contemporary artery-first approaches in pancreatoduodenectomy. *Br J Surg*. 2023;110(12):1570-1573. doi:10.1093/bjs/znad175

6.	Kinny-Köster B, van Oosten AF, Habib JR, et al. Mesoportal bypass, interposition graft, and mesocaval shunt: Surgical strategies to overcome superior mesenteric vein involvement in pancreatic cancer. *Surgery*. 2020;168(6):1048-1055. doi:10.1016/j.surg.2020.07.054

7.	Diener MK, Mihaljevic AL, Strobel O, et al. Periarterial divestment in pancreatic cancer surgery. *Surgery*. 2021;169(5):1019-1025. doi:10.1016/j.surg.2020.08.030

8.	Habib JR, Kinny-Köster B, van Oosten F, et al. Periadventitial dissection of the superior mesenteric artery for locally advanced pancreatic cancer: Surgical planning with the ''halo sign'' and ''string sign''. *Surgery*. 2021;169(5):1026-1031. doi:10.1016/j.surg.2020.08.031

9.	Loos M, Kester T, Klaiber U, et al. Arterial Resection in Pancreatic Cancer Surgery: Effective After a Learning Curve. *Ann Surg*. 2022;275(4):759-768. doi:10.1097/SLA0000000000004054

10.	Tee MC, Krajewski AC, Groeschl RT, et al. Indications and Perioperative Outcomes for Pancreatectomy with Arterial Resection. *J Am Coll Surg*. 2018;227(2):255-269. doi:10.1016/j.jamcollsurg.2018.05.001

11.	Bachellier P, Addeo P, Faitot F, Nappo G, Dufour P. Pancreatectomy With Arterial Resection for Pancreatic Adenocarcinoma: How Can It Be Done Safely and With Which Outcomes?: A Single Institution's Experience With 118 Patients. *Ann Surg*. 2020;271(5):932-940. doi:10.1097/SLA.0000000000003010

12.	Nakamura T, Okada KI, Ohtsuka M, et al. Insights from managing clinical issues in distal pancreatectomy with en bloc coeliac axis resection: experiences from 626 patients. *Br J Surg*. 2023;110(10):1387-1394. doi:10.1093/bjs/znad212

13.	Stoop TF, Bergquist E, Theijse RT, et al. Systematic review and Meta-analysis of the Role of total Pancreatectomy as an Alternative to Pancreatoduodenectomy in Patients at High Risk for Postoperative Pancreatic Fistula: is it a Justifiable Indication? *Ann Surg*. 2023;278(4):e702-e711. doi:10.1097/SLA.0000000000005895

14.	Suurmeijer JA, Emmen AM, Bonsing BA, et al. Nationwide validation of the ISGPS risk classification for postoperative pancreatic fistula after pancreatoduodenectomy: "Less is more". *Surgery*. 2023;173(5):1248-1253. doi:10.1016/j.surg.2023.01.004

15.	Del Chiaro M, Segersvard R, Rangelova E, et al. Cattell-Braasch Maneuver Combined with Artery-First Approach for Superior Mesenteric-Portal Vein Resection During Pancreatectomy. *J Gastrointest Surg*. 2015;19(12):2264-2268. doi:10.1007/s11605-015-2958-1

16.	Heckler M, Polychronidis G, Kinny-Koster B, et al. Thrombosis and anticoagulation after portal vein reconstruction during pancreatic surgery - A systematic review. *J Gastrointest Surg*. 2025;29(1):101852. doi:10.1016/j.gassur.2024.10.007

17.	Marchetti A, Garnier J, Habib JR, et al. The APROVE (Anti-coagulation/Platelet Treatment in Pancreatic Resections Involving Vascular Reconstruction) Study: Results from a Worldwide Survey. *Ann Surg Oncol*. 2025;32(10):7400-7409. doi:10.1245/s10434-025-17686-y

18.	van Veldhuisen E, Vogel JA, Klompmaker S, et al. Added value of CA19-9 response in predicting resectability of locally advanced pancreatic cancer following induction chemotherapy. *HPB (Oxford)*. 2018;20(7):605-611. doi:10.1016/j.hpb.2018.01.001

19.	Heger U, Sun H, Hinz U, et al. Induction chemotherapy in pancreatic cancer: CA 19-9 may predict resectability and survival. *HPB (Oxford)*. 2020;22(2):224-232. doi:10.1016/j.hpb.2019.06.012

20.	Dekker EN, van Klaveren D, Verkolf EMM, et al. Carbohydrate antigen 19-9 (CA19-9) response after induction FOLFIRINOX for locally advanced pancreatic cancer identifies patients who may benefit from surgical exploration: multicentre, observational cohort study. *Br J Surg*. 2025;112(2):znaf011. doi:10.1093/bjs/znaf011

21.	Rompen IF, Sereni E, Habib JR, et al. Development of a Composite Score Based on Carbohydrate Antigen 19-9 Dynamics to Predict Survival in Carbohydrate Antigen 19-9-Producing Patients With Pancreatic Ductal Adenocarcinoma After Neoadjuvant Treatment. *JCO Precis Oncol*. 2024;8:e2400193. doi:10.1200/po.24.00193

22.	Dutch Pancreatic Cancer Group. Flowdiagram postoperatieve diarree na pancreatectomie met arteriële divestment. Accessed at December 25, 2024. Available at: https://dpcg.nl/wp-content/uploads/2022/05/Flowdiagram-Postoperatieve-diarree-na-pancreatectomie-met-arteriele-divestment.pdf

23.	Stoop TF, Croce C, Ishida H, et al. Neurogenic diarrhoea after superior mesenteric artery divestment or resection for pancreatic adenocarcinoma following preoperative chemotherapy: international multicentre retrospective study. *BJS Open*. 2026; doi:10.1093/bjsopen/zrag057

24.	Conroy T, Pfeiffer P, Vilgrain V, et al. Pancreatic cancer: ESMO Clinical Practice Guideline for diagnosis, treatment and follow-up. *Ann Oncol.* 2023;34(11):987-1002. doi:10.1016/j.annonc.2023.08.009
